# The Tuning of Human Motor Response to Risk in a Dynamic Environment Task

**DOI:** 10.1371/journal.pone.0125461

**Published:** 2015-04-22

**Authors:** Amber Dunning, Atiyeh Ghoreyshi, Matteo Bertucco, Terence D. Sanger

**Affiliations:** 1 Department of Biomedical Engineering, University of Southern California, Los Angeles, California, United States of America; 2 Department of Neurology, University of Southern California, Los Angeles, California, United States of America; 3 Department of Biokinesiology, University of Southern California, Los Angeles, California, United States of America; 4 Children’s Hospital of Los Angeles, Los Angeles, California, United States of America; University Medical Center Groningen UMCG, NETHERLANDS

## Abstract

The role of motor uncertainty in discrete or static space tasks, such as pointing tasks, has been investigated in many experiments. These studies have shown that humans hold an internal representation of intrinsic and extrinsic motor uncertainty and compensate for this variability when planning movement. The aim of this study was to investigate how humans respond to uncertainties during movement execution in a dynamic environment despite indeterminate knowledge of the outcome of actions. Additionally, the role of errors, or lack thereof, in predicting risk was examined. In the experiment, subjects completed a driving simulation game on a two-lane road. The road contained random curves so that subjects were forced to use sensory feedback to complete the task and could not rely only on motor planning. Risk was manipulated by using horizontal perturbations to create the illusion of driving on a bumpy road, thereby imposing motor uncertainty, and altering the cost function of the road. Results suggest continual responsiveness to cost and uncertainty in a dynamic task and provide evidence that subjects avoid risk even in the absence of errors. The results suggest that humans tune their statistical motor behavior based on cost, taking into account probabilities of possible outcomes in response to environmental uncertainty.

## Introduction

Humans live in an environment that is constantly risky. In this study, we describe risk specifically as a combination of two factors: the probability of failure and the cost of failure [1]. A high cost of failure but low probability is not generally considered risky (standing several meters away from the edge of a cliff). Likewise, a high probability of failure but low cost is not regarded as risky (standing on the edge of a step). It is only where high likelihood of failure converges with high cost, when we stand on the edge of the cliff, that we venture into high risk.

Previous studies [2–5] have investigated the effect of risk on motor planning. Trommershauser and colleagues [2] have demonstrated that humans are able to maximize expected gain by using internal representations of the magnitude of outcome uncertainty. When outcome uncertainty was artificially enhanced by randomly perturbing trajectory end-points, subjects still demonstrated the ability to maximize reward based on end-point variability by shifting their mean trajectory endpoints in response to changes in penalties and location of the penalty region relative to the target region. Furthermore, it has been shown that subjects respond to changes in uncertainty when it is artificially increased or decreased without cue during an experiment. However, these experiments all investigate behavior in discrete-tasks, such as rapid pointing to a target. While there is a general lack of consensus on the degree of online error correction during motor program execution involved in these rapid movements, their duration is certainly too short to take full advantage of feedback control loops [6], [7] and therefore they rely primarily on motor planning [8]. In such experiments, we can investigate the effect of uncertainty on motor planning but not the effect on ongoing control of continuous movements.

While there is good evidence that humans plan movements taking risk into account, it is not clear how this occurs. For example, people might avoid actions that have previously led to poor outcomes as predicted by error-driven learning [9]. We consider the hypothesis that humans actively and continuously estimate both the probability of failure and the cost of failure, and that they make ongoing corrections to movement based on these estimates. In general, the probability and cost of failure may vary throughout the workspace, so to do this requires maintaining estimates of these values for all states that could possibly result from movement errors. This ability is a foundation of risk-aware control, a theory of motor control in humans that links ideas in optimal control with existing literature on risk behavior in humans [1]. If humans have this ability, then it is also possible to estimate risk without experiencing failure. Therefore we hypothesize that humans will respond to perceived risk even in situations where failure has not been experienced. At the most extreme, this means that humans will select movements that reduce risk even when the probability of failure is negligible.

**Fig 1:**
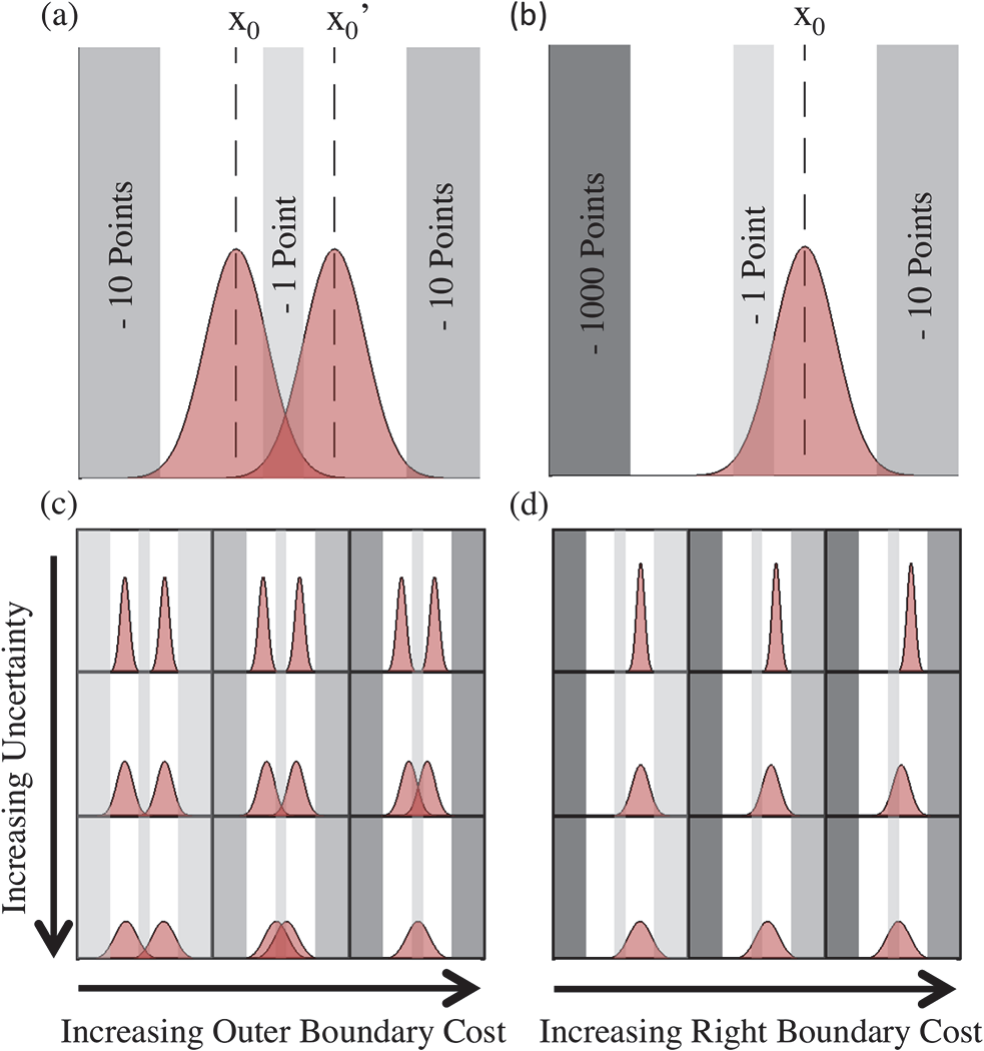
Theoretical minimization of cost under uncertainty. In Fig 1 (a)-(d), the shaded red distributions represent an uncertainty or variability in position. Grey bars signify penalty regions, the darker the grey, the higher the cost. The peaks of the curves illustrate the optimal position to minimize cost based on the standard deviation of uncertainty and the cost function. In (a) the loss function is symmetrical. The result is that there are two optimal positions that will minimize cost. Fig 1 (c) demonstrates the effect of increasing the cost of the outer boundary, dark grey regions, from left to right (1, 10, 100). The result is a shift in peaks toward the lower cost region in the center. Similarly, as the standard deviation of uncertainty increases from top to bottom (.35,. 75, 1) the optimal position again shifts toward the center lower cost region. At high standard deviation of uncertainty and high outer boundary cost, the optimal position becomes directly in the center of the middle region. Fig 1 (b) and (d) illustrate the same phenomenon for an asymmetrical loss function. Here the left boundary penalty remains very high (1000 points) while the right boundary in (d) increases from left to right (1, 10, 100). In this case there are no longer two optimal positions, only one in the segment that is farther away from the high cost.

To test this hypothesis, we designed a driving simulation experiment with a cost function similar to that of Fig 1. Each lane became a reward region and driving off the road or between lanes resulted in a point penalty. If humans maintain estimates of both probability of failure and cost of failure, then where in a lane the subject drives should depend on the specific form of the cost function. We further predict that these changes in behavior do not require subjects to experience failure (driving off the road).

## Materials and Methods

### Subjects

Twelve naïve subjects, ages 22 to 35, 9 males and 5 females, participated in the experiment. The University of Southern California Institutional Review Board approved the study protocol. All subjects gave informed written consent for participation and received compensation in proportion to their final score plus a base sum (Study IRB# UP_09_00263). Authorization for analysis, storage, and publication of protected health information was obtained according to the Health Information Portability and Accountability Act (HIPAA).

### Apparatus

The experiment was performed on an iPad2 (iOS 6.0, resolution of 1024x740 pixels) in landscape orientation. A custom application was created using CoronaSDK (Version 2012.11.15. Palo Alto, California: Corona Labs Inc., 2012). The update rate of the screen and rate of data acquisition was 30 fps.

### Stimuli and Procedure

The experiment took approximately one hour to complete, with small breaks as necessary, and was completed in a dimly lit room to avoid screen glare. For biomechanical uniformity, subjects were instructed to sit in a chair, maintaining their shoulders against the backrest, and to keep their elbows at approximately 90 degrees with their biceps inline with their torso during the entire experiment. Subjects grasped the sides of the screen with both hands at all times. Instructions for the experiment were verbally specified by the experiment administrator and presented again on the iPad screen for the subjects to read once they entered the application.

**Fig 2:**
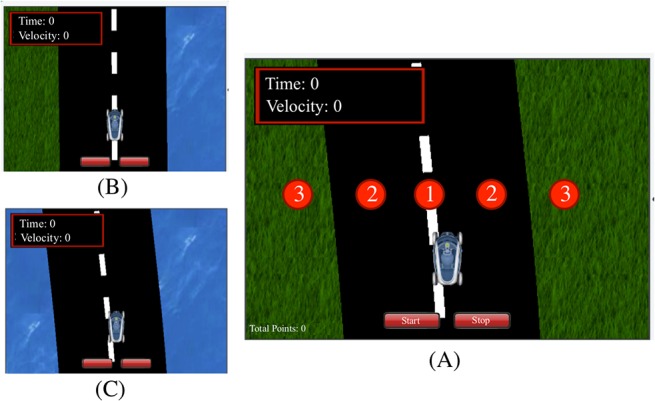
iPad application screen view. The subjects pressed the red start button to begin each trial (and were asked to not press the stop button during any trial). The time and velocity of the car was provided in the upper left hand corner of the screen. The three regions of speed are labeled in the figure with circles. Region 1 produced acceleration to maximum speed of 1100 pixels/sec; region 2 decelerated the car to 550 pixels/sec; region 3 immediately stopped the car to 2 pixels/sec. Cost functions: (A) symmetric low-cost, (B) asymmetric, (C) symmetric high-cost.

In the experiment, subjects maintained one-dimensional “steering” control of a vehicle in a driving simulation. The goal of the game was to complete each trial as quickly as possible, where the speed of the car was determined solely by position on a two-lane road. While on the road, driving within a lane yielded acceleration to the maximum velocity (1100 pixels/sec), driving on the dashed line between the two lanes caused the vehicle to decelerate to 550 pixels/sec, and hitting the grass along the side of the road slowed the car to 2 pixels/sec (which will be referred to as “stopped” as the car could hardly be detected as moving). Fig 2 contains a screenshot of the application. Subjects were able to control the position of the car by tilting the iPad in the left/right directions. Points awarded were inversely proportional to the time taken to complete each trial. Subjects could earn a maximum of 100 points per trial if they maintained the maximum velocity along the entire length of the road and could not earn less than 0 points due to speed penalties. Implementing the cost function in this manner effectively reinforced the cost, since more successful trials were linked not only to increased points and therefore increased monetary reward, but also decreased experiment time.

In addition to inherent motor variability, uncertainty was artificially enhanced by corrupting the responses of the subject with random, Gaussian-distributed horizontal perturbations at a frequency of 30Hz (the same frequency as the screen updates). Within the context of this study we will define this imposed variability as motor noise. The effect was similar to the sensation of driving on a bumpy road; the subject was able to determine the present car position, but was uncertain exactly where they may be in the next instant. Thus the effect of the motor noise was to increase uncertainty of future position and alter the probability of failure. It is important to note, however, that this is not identical to driving on a bumpy road, where noise is dependent on position on the road. The noise was generated at a constant time interval so that slowing down would not make the task significantly easier. There were five levels of imposed motor noise: 0 (no additional noise), 4, 8, 12, and 16 pixels standard deviation (psd). Each trial was 30,000 pixels in length and took approximately 30 to 60 seconds to complete. The first 10,000 pixels of each trial were practice, giving the subject enough time to get up to speed and adjust to the noise level, during which no points were accumulated or lost. The car always started a trial where it ended in the previous, unless it was the first trial of a block, then the car started in the middle of the road. The road was 500 pixels wide, the center dashed line was 15 pixels wide, and the car width was 40 pixels. The curves of the road were generated using Bezier curves [10] with random anchor points derived from a uniform distribution.

During the experiment, subjects’ responses were tested to three cost functions, blocked into two sets of trials: block A) the symmetric low-cost and block B) the asymmetric and symmetric high-cost. In block A, subjects completed a random sequence of the 5 uncertainty levels 3 times, for a total of 15 trials using the cost function as described above (grass on both sides). During block B, water replaced the grass on one or both sides of the road respectively. Running into the water caused an immediate stop and replaced the car to the center of the road (the timer was stopped so that this was equivalent with respect to time to running into the grass), but with an additional 500-point penalty. In environments with water there were only 4 degrees of additive noise (0, 4, 8, and 12 psd) as during pilot testing the highest noise level caused subjects to generally earn very negative points and discouraged subjects from heeding the point system. Therefore, in order to maintain a high sensitivity to risk, we did not include a noise level of 15 psd in environments with water. Each noise level was repeated twice with water on both sides and twice with water on one side (counter-balanced) for a total of 16 trials in a pseudo-random sequence.

Each subject first learned control of the car in the low-cost environment during 15 practice trials (same as block A). Each subject was informed that driving on the black part of the road would yield maximum velocity, while touching the white center lane would cause the car to slow down and hitting the side of the road would bring the car to a stop. Subjects were also told that they could earn a maximum of 100 points per trial and were encouraged to explore the road during the practice block during which points earned or lost would not count towards their monetary reward. After finishing the practice block and brief rest, subjects then completed block A and block B in random order with rest in between.

The raw data collected from subjects has been made available as a supporting file (S1 Dataset).

### Data analysis

During the experiment we recorded position of the car and the time it took to complete each trial. Points were recorded, but not used in analysis as they were rounded, and therefore less accurate, and only piecewise proportional (subjects could not earn less than 0 points from speed penalties). Trial time did not reflect the effects of falling into the water, but only one subject incurred this penalty.

All analysis was done in MATLAB (version 7.13.0.564. Natick, Massachusetts: The MathWorks Inc., 2011) and R: A Language and Environment for Statistical Computing (version 3.0.1. Vienna, Austria: R Development Core Team, 2013). Position data for subjects was pooled to represent average behavior of the sample population and fit to Eq 1 using maximum likelihood estimation. In these functions, zero is center of the road and units are in pixels.

$$y=(p)f_{1}(x|\mu_{1},\sigma_{1})+(1-p)f_{2}(x|\mu_{2},\sigma_{2})$$ (1)

$$f(x)=\frac{1}{\sigma \sqrt{2\pi }}e^{\frac{-{\left(x-\mu \right)}^{2}}{2\sigma^{2}}}$$

In Eq 1, x is position, μ_1_ and μ_2_ are the means of each Gaussian, σ_1_ and σ_2_ are the standard deviation of the respective Gaussians, p is the weighting factor between the Gaussians, and y is the resulting probability of position. The resulting parameters, μ_1_, μ_2_, σ_1_, σ_2_, and p, were interpolated (using cubic spline interpolation) across motor noise to generate estimated continuous position probability distributions for each cost function.

Additionally, the position data for each subject, for each cost function at each noise level were fit to Eq 1 using maximum likelihood estimation. In order to quantify the average distance from the center of the road that subjects attempted to maintain for each condition, the absolute value of μ_1_ and μ_2_ were weighted by the area under each Gaussian, p and p-1, and summed. Linear regressions were fit to the means across subjects of the four lowest levels of motor noise of each task condition.

A two-way ANOVA was performed using the aov function of the R statistical computing environment. The R model aov(Position ~ Uncertainty * Task) was used to test the differences in distance from the center of the road between the level of motor noise and task type. In this model, uncertainty is the quantifiable level of simulated motor noise imposed, and task was the type of environmental cost function. Post hoc pairwise comparisons were made between each of the three cost functions within each uncertainty level using paired-t tests with an alpha value of 0.05.

A two-way repeated measures ANOVA was also performed to test the differences in trial time between the level of motor noise and task type. The test was performed in R using the model aov(Time ~ Uncertainty * Task + Error(Subject)). Again, paired-t tests were used to make post hoc pairwise comparisons of the difference between each of the three cost functions within each uncertainty level.

We were also interested in the role that errors played in forming behavior. Therefore, the percentage of failed trials, trials in which the subject went outside the road, was calculated for each level of motor noise of the low-cost task and asymmetric task. (It is not presented for the symmetric high-cost task, because only one such failure occurred amongst all subjects in this environment.)

## Results

**Fig 3:**
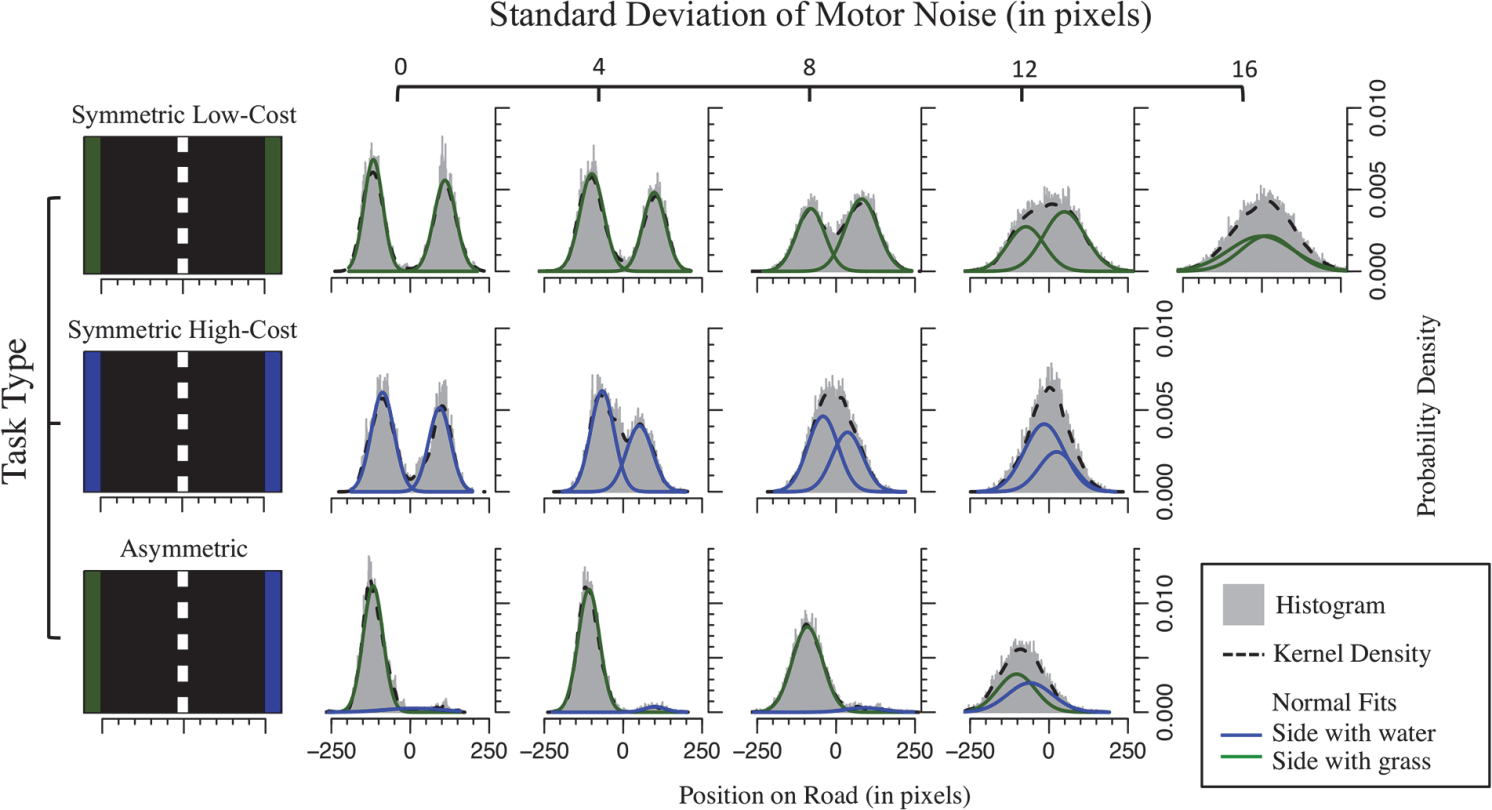
Raw Population position data. Plots are the histograms of the pooled subject data for each task type (by row) and uncertainty level (by column). The dashed lines are the kernel densities of the data and the solid lines are the bimodal Gaussian fits (see methods). Green lines represent the position Gaussian near grass (low-cost) while blue represents a position peak near water (high cost). In the asymmetric task, the bottom row, it can be seen that subjects maintained a position far away from the side with water. The x-axis represents the position of the center of the car on the road in pixels. (The road is 500 pixels wide, and the car is 40 pixels, so the subject ran off the road at ±230 pixels.) These images depict a trend similar to Fig 1. As the outer boundary costs increased, the subjects moved toward the center of the road. Similarly, as the standard deviation of uncertainty increased, subjects also moved toward the center of the road.

**Fig 4:**
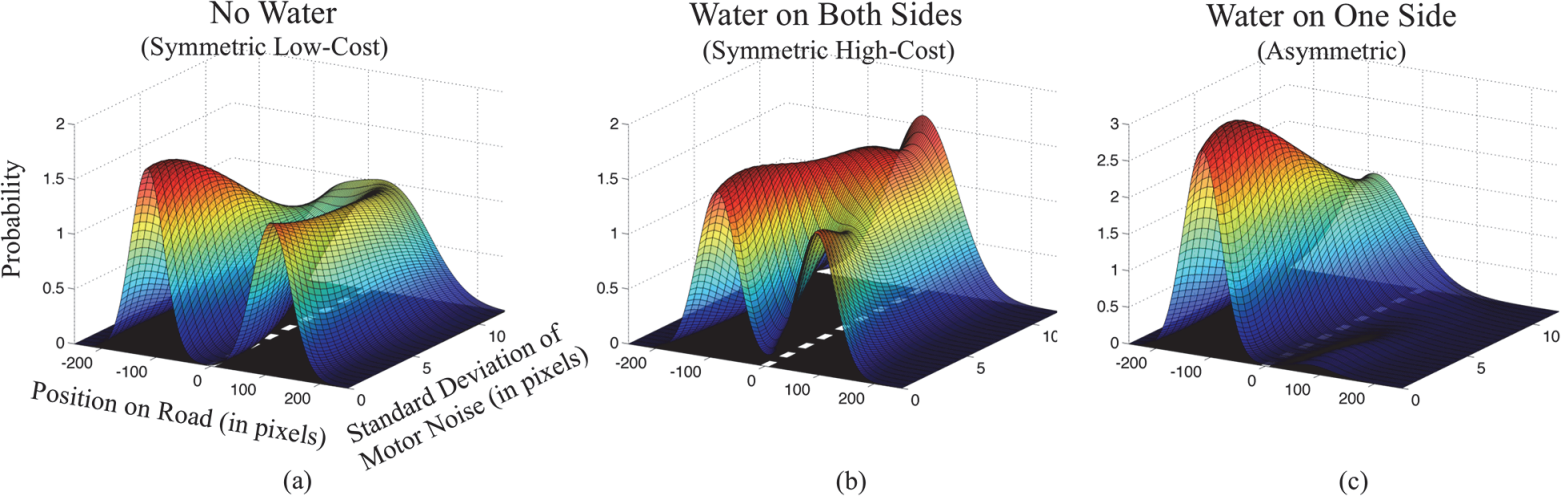
Continuous position probability distribution as a function of uncertainty. Variables of bimodal Gaussian fits (μ1, μ2, σ1, σ2, and p) from Fig 3 were interpolated (using cubic spline interpolation) across noise levels. This demonstrates an estimate of the probability of where on the road a subject will be at any given instant as a function of motor noise.

In order to quantify any learning effect within the course of the experiment, distance from the center of the road for each condition from subjects who completed block A first were compared with those of subjects who completed block B first. They were not significantly different (p < 0.05), therefore it was concluded that after the initial practice trials, there was no observable learning effect. As expected, position data resemble bimodal Gaussian distributions as shown in Fig 3. As motor noise increases, the two peaks of the distribution tend toward each other, merging into a single normal distribution at high motor uncertainty. Essentially, subjects reacted accordingly to motor noise; they stayed within a lane at low levels of uncertainty and moved toward the center of the road at high levels of uncertainty, illustrated in Fig 4. This reflects a tradeoff in which they accept the higher cost of driving on the median in order to avoid the risk of driving off the road. In the asymmetric risk environment, position data appropriately reflects the asymmetric cost with highly disproportionate peaks, so that subjects have a strong tendency to drive on the side of the road that is farthest from the water. However, as the noise increases, subjects drive closer to the middle of the road and thus closer to the water in order to balance the risk of falling to either side. Table 1 contains the percentage of time that subjects spent in the lane near the grass (away from the water) during this task.

**Table 1:** Percentage of time spent in the lane farthest from water in the asymmetric cost environment.

Standard Deviation of Motor Noise (in pixels)	0	4	8	12
Percentage of Time Spent in Lane Away From Water	96.12%	96.23%	91.53%	88.87%

Subjects spent significantly more time in the lane opposite of the water. The increase in percentage of time with increased motor noise can be attributed to subjects moving closer to the center of the road to avoid hitting the grass. As they moved toward the center of the road, they crossed the centerline into the lane adjacent to the water more often, albeit still briefly.

**Fig 5:**
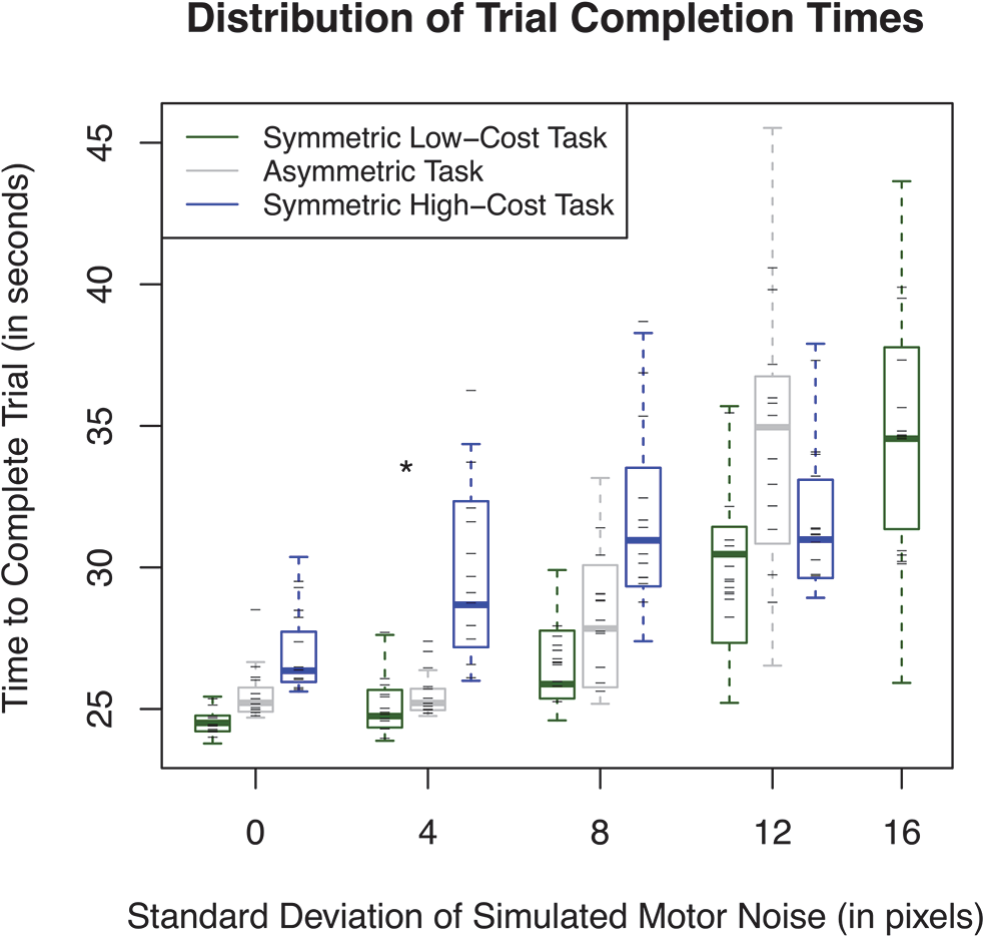
Box plot of subjects’ average trial times. Dashes indicate the average time each subject took to complete that task. Notice in general the distribution is greater for higher risk tasks indicating the differences in subjects’ risk awareness. The increase in distribution with increased motor noise demonstrates that some subjects were more adept than others at playing the game. The asterisk indicates the only pair of trial times that were not significantly different.

Over the four lowest levels of uncertainty, subjects took an average of 26.69 seconds to complete a trial in the symmetric low-cost environment, 28.51 seconds in the asymmetric environment, and 30.30 seconds in the symmetric high-cost environment. A two-way repeated measures ANOVA showed that there was a significant effect of motor noise [F(4,408) = 102.469, p < 0.001], task type [F(2,408) = 43.892, p < 0.001], and interaction [F(6,408) = 6.417, p < 0.001] on the time it took to complete a trial. This is not especially informative since the implemented cost function directly affects trial completion time; increased motor noise will lead to larger trial times regardless of how the subject responds. The more interesting conclusions lie in the post hoc pairwise comparisons between task types at equal levels of uncertainty. Of the twelve comparisons, all pairs except one were significantly different (p < 0.05), the symmetric low-cost task and the asymmetric task at 4 psd motor noise. In other words when risk was introduced into the environment, subjects sacrificed time and points to steer clear of the high cost regions, shown in Fig 5. Comparing the symmetric tasks, there is a shift in the y-intercept of the regressions, but the slopes are almost identical. This indicates that there is a constant effect of the increased cost on subjects’ responses independent of motor noise (at least within this range).

**Fig 6:**
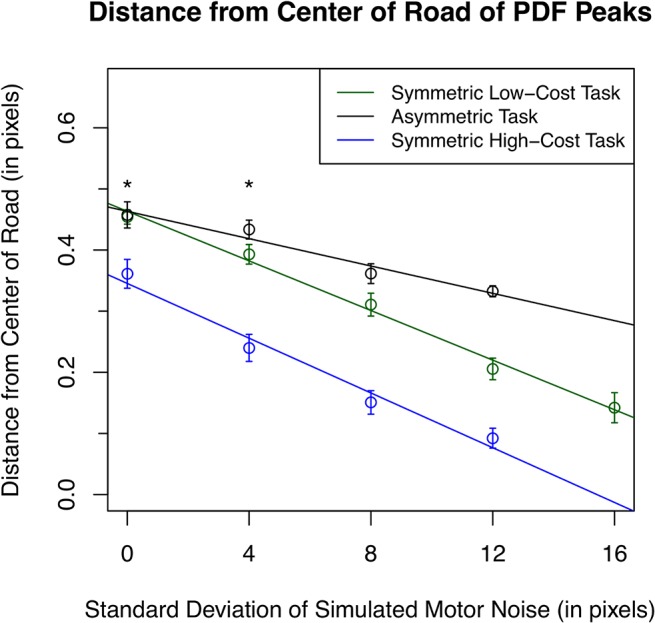
Regression on the distance from the center of the road subjects maintained vs. level of motor noise. Points indicate the mean distance from the center of the road of all subjects derived from the peaks of the fitted probability density functions (see methods) for each task type and uncertainty level. As motor noise increased, subjects’ position shifted proportionally toward the center of the road. Position is normalized to 250 pixels so 0 is the center of the road and 1 is the edge of the road. Errors bars indicate the standard error of subjects. Solid lines represent the linear regressions fit for each task type. Asterisks indicate the pairs of values with insignificant differences.

The mean distance from the center of the road, calculated from the parameters fit to Eq 1 as explained in methods, for each condition can be seen in Fig 6. Over the four lowest levels of uncertainty, the mean distance from the center of the road (normalized to the road width) was 0.3408 (SE ±.0157) for the symmetrical low-cost task, 0.3962 (SE ±.0108) for the asymmetrical task, and 0.2110 (SE ±.0178) for the symmetrical high-cost task. Linear regressions demonstrate a linear dependency of distance from the center of the road on uncertainty level [R^2^ (symmetrical low-cost task) = 0.982, R^2^ (symmetrical high-cost task) = 0.984, R^2^ (asymmetrical task) = 0.945]. A two-way ANOVA showed that there was a significant effect of motor noise [F(4,408) = 92.64, p < 0.001], task type [F(2,408) = 112.61, p < 0.001], and interaction [F(6,408) = 4.157, p < 0.001] on the distance of the bimodal Gaussian position distribution peaks from the center of the road. In post hoc pairwise comparisons between task types within each uncertainty level, all were significant (p < 0.05) except between the symmetric low-cost task and asymmetric task at 0 and 4 psd noise. Details can be found in Fig 6.

**Fig 7:**
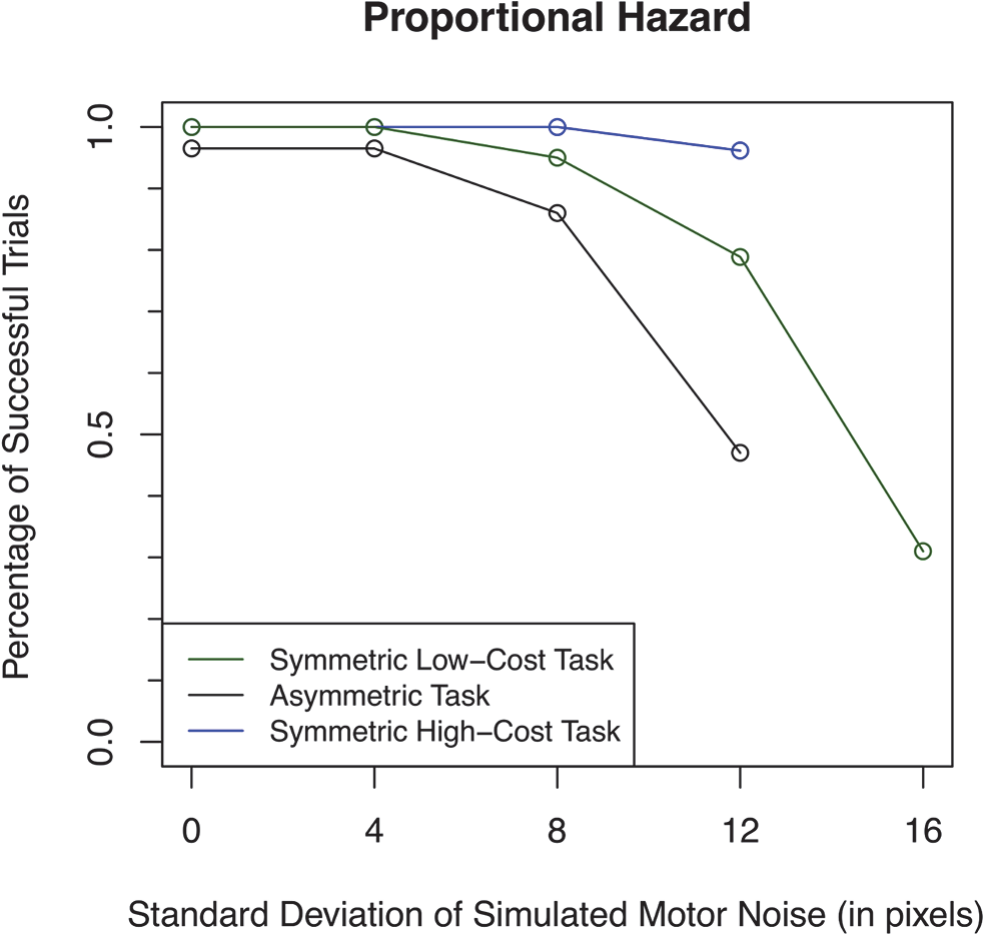
Proportional hazard model of successful trials. Points indicate total percentage of successful trials for all subjects at each level of uncertainty, where a successful trial is defined as a trial during which the subject never ran off the road. (The green line represents the symmetric low-cost task, the black line is the asymmetric task, and the blue line indicates the symmetric high-cost task.) At all uncertainty levels, failed trials occurred more than twice as often in the asymmetric task than in the low-cost task. That is that subjects stayed so far away from the water that they hit the grass on the opposite side of the road much more frequently.

Subjects, on average, ran off the road in more than twice as many trials at every level of uncertainty in the asymmetric task than in the low-cost task, shown in Fig 7. These numbers do not include failures during practice. However, at the two lowest levels of motor noise in the low-cost task, even during the initial practice block, no subject ran off the road. Additionally, only one subject ever fell off the road in the high-cost task. This demonstrates that subjects react to the probability of failure even when failure has not been experienced. This observation is inconsistent with an adaptive reduction in error, and instead must represent a mechanism that estimates and predicts failure that has not yet occurred.

## Discussion

It has been previously suggested that humans act as Bayes optimal observers in motor planning tasks, such as rapid pointing, by modifying behavior to compensate for uncertainty [11–17]. In this study we were interested in investigating if this behavior extended to response to cost and uncertainty in a continuous task controlled with feedback, and if this behavior could be done without experiencing error in the task.

At no additional motor noise, subjects on average maintained a bimodal Gaussian distribution near the center of either lane (approximately 10 pixels closer to the center line than the road boundary) in the low-cost environment. This shows that qualitatively optimal behavior could be performed with a bimodal cost function that is more complicated than the single target used in most prior studies. In the asymmetric environment, subjects stayed in the lane closer to the grass more than 95% of the time, and subsequently treated this lane almost identically to the low-cost task. In the symmetric high-cost environment, subjects moved more than an additional 20 pixels towards the center of the road compared to the symmetrical low-cost environment at equal uncertainty levels. Subjects shifted their behavior in the presence of risk even though no subject left the boundaries of the road in the symmetrical low-cost task at 0 and 4 psd motor noise. Based on the observation of error, there is in fact no reason to pull away from the side of the road when the cost to running off the road was increased. This behavior is either suboptimal or optimal with respect to an internally derived cost function that does not match the empirical data. This suggests that predictions of failure not only carry very long tails, but predict possible error even when none has previously occurred. Additionally, only one subject ever fell into the water, but every subject still demonstrated a significant shift in behavior in the symmetric high-cost environment. This is significant since the common model for learning in motor control is error-driven learning, and this observation suggests that human performance is often not driven by errors. This demonstrates that subjects made predictions of both the likelihood and cost of failure, and our results are consistent with the existence of internal estimates of probability of failure and cost of failure.

As the uncertainty increased, subjects adjusted their position more towards the center of the road. The distance subjects moved away from the road boundary was dependent on the motor noise at least within the constraints of this task. While subjects behaved similarly in the asymmetric task to the symmetrical low cost-task at low levels of uncertainty, subjects adjusted their behavior differently at high levels of uncertainty. Subjects did not move towards the center of the road as much in the asymmetric task in order to avoid running over the centerline and into the high-cost region (water) on the opposite side. This occurred even though it caused subjects to hit the low-cost region (grass) much more often and meant taking significantly longer to complete the asymmetric task at the highest noise level than either of the other two tasks, see Fig 5. However, since only one subject ever hit the water in any symmetrical high-cost environment, this again demonstrates that for most subjects this shift in behavior was not necessary and shows how sensitive humans are to high-risk regions.

It is important to recognize that the concept of risk we describe in this paper, “risk-awareness”, is derived from risk-aware control and a fundamentally different concept than the more ubiquitous “risk-sensitivity” originating from an economical decision-making perspective of motor control [1,18]. Risk-sensitivity is used to describe inter-individual differences in response to risk, where risk is defined in terms of higher moments of reward. In this experiment, an explicit cost function is provided so there should not be much inter-individual difference. In the context of this paper we are defining awareness of risk as continuous estimates of both the cost of failure and probability of failure in a task.

Unlike previous studies, we did not compare subjects’ responses to the “optimal” response. It has already been demonstrated that in navigating 2-dimensional terrains humans’ behavior is typically suboptimal [19]. It is certainly feasible to create a cost function sufficiently obscure or complicated to prevent humans from responding optimally. And there are many other considerations such as attention, fatigue, motivation, etc. that are impossible to quantify and implement in the estimation model, but that certainly affect the complete cost function a subject would theoretically minimize. Additionally, the results suggest that subjects are tuning their behavior to a probability function that is a result of both pre-existing assumptions about variability and measurements of the empiric variability of the task. Because we do not know the assumptions a subject makes of the underlying probability distribution, whether subjects are maximizing expected utility correctly and the appropriateness of the assumptions are not completely discernable with this study. It can be concluded, however, that subjects are responding to the increase of risk in the task. So it was not the focus of this study to determine how closely humans are able to reproduce the optimal response in continuous tasks, but whether they demonstrate on-going awareness of risk.

Humans are relatively fragile creatures. Only through constant vigilance and avoidance of risk do we remain safe from injury. We have shown that not only do we consider risk when initially planning a movement, but also that we are constantly evaluating the environmental cost function. Moreover, we are constantly making predictions of failure, even in cases where we have never experienced that failure. Our survival depends on knowing that falling off the cliff is going to be unpleasant without having to experience it first.

## Supporting Information

S1 Dataset Raw Data. Data is organized first by subjects (1–12). Data is then subcategorized by environment (grass/grass grass/water and water/water) and trial number. For each trial, the dataset includes time to complete each trial in msec and position normalized to the center of the road for each trial recorded at ~33msec.
(TXT)
